# Magnesium Sulfate in combination with Nifedipine in the treatment of Pregnancy-Induced Hypertension

**DOI:** 10.12669/pjms.36.2.706

**Published:** 2020

**Authors:** Cuiping Xiang, Xuegui Zhou, Xiaoxia Zheng

**Affiliations:** 1 Cuiping Xiang. Department of Obstetrics, Binzhou People’s Hospital, Shandong, 256610, China; 2 Xuegui Zhou. Department of Obstetrics, Binzhou People’s Hospital, Shandong, 256610, China; 3 Xiaoxia Zheng Department of Gynecology Binzhou City Center Hospital, Shandong, 251700, China

**Keywords:** Magnesium sulfate, Nifedipine, Pregnancy induced hypertension syndrome

## Abstract

**Objective:**

To investigate the effect of magnesium sulfate combined with nifedipine in the treatment of pregnancy-induced hypertension syndrome (PIHS).

**Methods:**

Total 118 pregnant women with PIHS who were admitted to our hospital from April 2017 to June 2018 were randomly divided into control group (59 cases) and observation group (59 cases). The observation group was treated by magnesium sulfate in combination with nifedipine, while the control group was treated by magnesium sulfate. The therapeutic effect, serum leukaemia inhibitory factor (LIF), Apelin level, blood pressure, blood viscosity, urinary protein, S/D and Umbilical Artery Resistance Index (UARI) were compared between the two groups.

**Results:**

The effective rate of the observation group was 94.9%, higher than 83.1% of the control group, and the difference was statistically significant (P<0.05). The decrease level of systolic and diastolic blood pressure in the observation group was better than that in the control group, and the difference was statistically significant (P<0.05). The decrease of blood viscosity, urinary protein, S/D and UARI in the observation group was greater than that in the control group, and the difference was statistically significant (P<0.05). The improvement of serum LIF and Apelin levels in the observation group was better than that in the control group (P<0.05), and the difference was statistically significant (P<0.05).

**Conclusion:**

Magnesium sulfate combined with nifedipine in the treatment of PIHS has a significant effect, which can effectively control edema, blood pressure, proteinuria and protect kidney. It is worth clinical promotion.

## INTRODUCTION

Pregnancy-induced hypertension syndrome (PIHS) mainly occurs after 20 weeks of pregnancy and in the early stage of puerperium. Causes of PIHS include placental ischemia, immunity decline and heredity of pregnant women.^1^-^3^ Its main clinical symptoms manifest as hypertension, edema, proteinuria, and even convulsions, coma, cerebrovascular accidents, placental abruption, fetal distress, intrauterine death and heart and kidney failure.^4^,^5^ The morbidity of PIHS is 9.4%^6^ in China and 1%~12% abroad.^7^ It can seriously affect the health of mothers and infants. It is the main cause of morbidity and death of mothers and perinatals in clinic. Therefore, effective treatment measures should be taken immediately after diagnosis. At present, the clinical commonly used magnesium sulfate monotherapy has been unable to achieve good results and cannot meet the actual needs of patients. In recent years, it has been found that magnesium sulfate combined with nifedipine can achieve good results.^8^,^9^ Nifedipine, a dihydropyridine calcium channel blocker, can effectively prevent calcium ions from entering the myocardium and smooth muscle for transmembrane transport and highly recognize it. Although it can hinder calcium ions from dispersing in the cells to some extent, it will not affect the concentration of plasma calcium ions.^10^,^11^ The combination of magnesium sulfate and nifedipine has positive significance in the treatment of PIHS. The purpose of this study was to explore the clinical effect of magnesium sulfate combined with nifedipine in the treatment of PIHS.

## METHODS

Total 118 pregnant women with PIHS who were admitted to our hospital from April 2017 to June 2018 were divided into control group and observation group by random color ball extraction method. The sample size can be determined using n=2*[(α+β)σ/δ]^2, where δ stands for the degree of distinction, σ stands for the overall standard deviation, α and β represent u values of α and β, and they can be referred to the row of degree of freedom υ=∞- in t critical value table. α can be unilateral or bilateral, but β only take unilateral value.

Pregnant women who satisfied the diagnostic criteria of Obstetrics and Gynecology^12^ and had clinical manifestations such as edema, urinary protein and hypertension after 20 weeks of pregnancy were included. Patients with severe heart, liver and kidney dysfunction, nephritis, diabetes mellitus, or essential hypertension were excluded. Fifty-nine patients in the observation group aged 21-42 years (average (29.8±3.1) years) had 24-36 weeks of gestation (average (29.5±4.6) weeks). There were 35 primipara and 24 multipara. There were 27 cases of mild pre-eclampsia, 21 cases of moderate pre-eclampsia and 11 cases of severe pre-eclampsia. Fifty-nine patients in the control group aged 22-39 years (average 29.2±4.1 years) and had 25-35 weeks of gestation (average (29.3±4.5) weeks). There were 33 primipara and 26 multipara. There were 25 cases of mild preeclampsia, 22 cases of severe preeclampsia and 12 cases of moderate preeclampsia. The results could be compared as there was no significant difference in clinical data between the two groups (P>0.05). This study was approved by the ethics committee (Ref.No. 116, Dated on December 24, 2018) of our hospital. All the selected patients signed the informed consent.

### Therapeutic method

Patients in the control group received conventional treatment such as sedation, diuresis, oxygen inhalation and volume expansion after admission and conventional nursing intervention and were guided patients to eat scientifically. 20 mL of 25% magnesium sulfate (Hangzhou Minsheng Pharmaceutical Co., Ltd., China; batch number: 20120627) was dissolved into 100 ml of 5% glucose solution and intravenously dripped. After 30 minutes, 40 ml of 25% magnesium sulfate was added into 500 ml of 5% glucose solution and intravenously dripped at 1.5 g/h. The dosage was maintained at a speed of 1-2 g/h. The concentration of magnesium sulfate injection was adjusted according to the disease condition and pressure of patients during treatment.

On the basis of the control group, patients in the observation group orally took 10 mg of nifedipine tablets (manufacturer: Shanxi Yunpeng Pharmaceutical Co., Ltd., China; SFDA approval number: H14020798; specification: 10 mg * 100 tablets), 3-4 times a day. The two groups were treated for one week.

### Observation indicators

The therapeutic effect, level of serum leukemia inhibitory factor (LIF), small molecular active polypeptide (Apelin) secreted by placenta tissue and blood pressure were observed. Blood viscosity, 24-hour urinary protein quantification, the ratio of fetal umbilical artery systolic pressure to diastolic pressure (S/D) and Umbilical Artery Resistance Index (UARI) were monitored regularly in the treatment period.

### Evaluation of curative efficacy

The clinical efficacies of patients with severe PIHS were compared between the two groups. The treatment was considered as significantly effective if the diastolic pressure and systolic pressure reduced to the normal levels, edema disappeared, and urine protein level returned to the normal range. The treatment was considered as effective if the diastolic pressure and systolic pressure significantly decreased after treatment and the edema and urine protein were significantly improved. The treatment was considered as ineffective if the blood pressure had no obvious decrease after treatment and the edema and urine protein had no obvious improvement. The formula of overall effective rate was: overall effective rate = (number of significantly effective cases + number of effective cases)/total number of cases × 100%.

### Statistical analysis

Data were processed using SPSS 20.0. Measurement data were expressed as Mean±SD; comparison between groups was performed using t test. Enumeration data were expressed as rate (%); comparison between groups was performed using Chi-square test. Difference was considered as statistically significant if P<0.05.

## RESULTS

The overall effective rate of the observation group was 94.9%, which was significantly higher than 83.1% in the control group; the difference was statistically significant (P<0.05, Table-I).

**Table-I:** Treatment effect between the two groups [n(%)].

Group	Observation group	Control group	X^2^	P
Significantly effective	33(55.9)	29(49.2)	/	/
Effective	23(39.0)	21(33.9)	/	/
Ineffective	3(5.1)	10(16.9)	/	/
Overall effective	56(94.9)	49(83.1)	7.895	<0.05

Before treatment, there was no significant difference in systolic and diastolic blood pressure between the two groups (P>0.05); the blood pressure of both groups after treatment decreased compared with that before treatment, and the decrease of systolic and diastolic blood pressure in the observation group was significant than that in the control group (P<0.05, Table-II).

**Table-II:** Prior-treatment and post-treatment blood pressure level between the two groups (mmHg).

Group		Observation group	Control group
Systolic blood pressure	Prior-treatment	110.54±10.42	111.55±10.24
	Post-treatment	88.23±6.54*^#^	99.87±6.87*
Diastolic blood pressure	Prior-treatment	171.25±15.55	170.26±15.36
	Post-treatment	125.42±10.57*^#^	139.58±10.22*

***Note:***

* means P<0.05 compared to prior-treatment;

# means P<0.05 compared to the control group.

Before treatment, there was no significant difference in the blood viscosity, urine protein, S/D and RI between the two groups (P>0.05). After treatment, the blood viscosity, urine protein, S/D and RI decreased in both groups. The decrease of blood viscosity, urine protein, S/D and RI in the observation group was greater than that in the control group (P<0.05), and the difference was significant (P<0.05, Table-III).

**Table-III:** Changes of blood viscosity, urine protein, S/D and RI.

Group		Observation group	Control group
Blood viscosity (m Pas)	Prior-treatment	4.73±1.13	4.84±1.22
	Post-treatment	2.22±0.53*^#^	3.27±0.96*
24 h urine protein quantity (g/24 h)	Prior-treatment	2.53±0.39	2.50±0.36
	Post-treatment	1.08±0.24*^#^	1.98±0.22*
S/D	Prior-treatment	2.73±0.31	2.74±0.32
	Post-treatment	1.79±0.33*^#^	2.45±0.38*
RI	Prior-treatment	0.59±0.05	0.57±0.06
	Post-treatment	0. 27±0.04*^#^	0.46±0.04*

***Note:***

* means P<0.05 compared to prior-treatment;

# means P<0.05 compared to the control group.

Before treatment, there was no significant difference in the serum LIF and Apelin levels between the two groups (P>0.05). After treatment, the improvement of the serum LIF and Apelin levels in the observation group was better than that in the control group, and the difference was statistically significant (P<0.05, Table-IV).

**Table-IV:** Prior-treatment and post-treatment serum LIF and Apelin levels between the two groups (ng/L).

Group		Observation group	Control group
LIF	Prior-treatment	688.69±31.23	684.54±30.54
	Post-treatment	759.58±30.55*^#^	711.98±31.25*
Apelin	Prior-treatment	384.54±25.62	389.54±26.66
	Post-treatment	220.57±21.86*^#^	279.56±22.88*

***Note:***

* means P<0.05 compared to prior-treatment;

# means P<0.05 compared to the control group.

## DISCUSSION

This study analyzed and compared the clinical effects of magnesium sulfate and magnesium sulfate in combination with nifedipine in the treatment of PIHS. The research results demonstrated that the overall effective rate of the observation group was higher than that of the control group, which was similar to the research results of Pasaribu et al.^13^ PIHS is a common gynecological and obstetric disease.^14^,^15^ The treatment for PIHS currently focuses on spasmolysis, pressure reduction, and cardiac load reduction, and magnesium sulfate is the preferred and main drug.^16^ As a spasmolytic drug, magnesium sulfate contains magnesium ions which can inhibit the release of acetylcholine from motor nerve-muscle junction to block the signal transduction at the nerve-muscle junction and alleviate muscle contraction, and it has a good effect in the treatment of eclampsia.^17^ It can also act on vascular smooth muscle to expand peripheral blood vessels, showing a strong antihypertensive effect, and can reduce blood pressure in a short time and relieve cardio-cerebral insufficiency in pregnant women.^18^ However, clinical practice shows that blood pressure is prone to rebound after drug withdrawal although magnesium sulfate alone can achieve a good control of blood pressure,^19^ which was not verified in this study as the follow up time was too short.

Nifedipine sustained-release tablet is a long-acting calcium antagonist. Its main function is to dilate the coronary artery, increase the blood flow of patients’ coronary artery, relax the smooth muscle inside the vessels, and achieve the goal of stabilizing the concentration of drugs.^20^ Nifedipine sustained-release tablets can also enter the transmembrane transport of myocardium and smooth muscle cells of patients through calcium ions to selectively inhibit the cells and relax the smooth muscle inside the blood vessels, thereby reducing blood pressure and systolic blood pressure.^21^,^22^ It is worth noting that nifedipine sustained-release tablet has significantly better antihypertensive effect compared to other angiotensin-converting enzyme inhibitors and is more safe compared to other angiotensin-converting enzyme inhibitors. For example, it will not affect the glucose metabolism; hence it has a significant therapeutic effect on hypertension patients with diabetes mellitus. A study has shown that magnesium sulfate combined with nifedipine can effectively promote smooth muscle relaxation, effectively reduce blood pressure and improve fetal nutrition.^23^ The results of this study also showed that the the decrease amplitudes of SBP and DBP of the observation group were more obvious than those of the control group, indicating that nifedipine combined with magnesium sulfate had significant effect in the treatment of PIHS and could effectively control blood pressure. This study also found that the plasma viscosity, proteinuria level, S/D and RI of the observation group were lower than those in the control group after treatment, indicating that nifedipine combined with magnesium sulfate could effectively relieve the symptoms of patients, which was consistent with the results of a previous study.^24^

LIF is a leukaemia inhibitory factor,^25^ and it can regulate the differentiation and proliferation of trophoblasts during pregnancy Apelin is a small molecule active polypeptide secreted by placenta tissue, which also has a bi-directional effect on blood pressure regulation in pregnant women. Both of the above two indicators are related to PIHS; hence they can be used as indicators for evaluating treatment means. The results of this study showed that the improvement of serum LIF and Apelin levels in the observation group was significantly better than that in the control group, indicating that the combination of the two drugs was effectively in regulating the serum LIF and Apelin levels and improve the therapeutic effect, which was also the novel result of this study compared to other literature.^26^

## CONCLUSION

In conclusion, nifedipine combined with magnesium sulfate in the treatment of PIHS has significantly better effective rate than intravenous drip of magnesium sulfate alone and can effectively control blood pressure, reduce plasma viscosity and urine protein quantity and regulate serum LIF and Apelin levels. It is worth clinical promotion. However, the follow-up time of this study is too short to follow up the pregnancy outcome of the patients, which needs follow-up study with large sample size in the future.

### Authors’ Contribution:

**CPX:** Study design, data collection and analysis, is responsible for integrity of research.

**XXZ & XGZ:** Manuscript preparation, drafting and revising.

**CPX & XGZ:** Review and final approval of manuscript.
